# Impact of climate change on the health of peruvians: challenges and strategies for a comprehensive response

**DOI:** 10.17843/rpmesp.2023.402.12998

**Published:** 2023-06-30

**Authors:** Luciana Blanco-Villafuerte, Stella M. Hartinger

**Affiliations:** 1 Latin American Center of Excellence in Climate Change and Health, Universidad Peruana Cayetano Heredia, Lima, Peru. Universidad Peruana Cayetano Heredia Latin American Center of Excellence in Climate Change and Health Universidad Peruana Cayetano Heredia Lima Peru

Climate change has a negative impact on the health of every society, including the Peruvian population. The effects of climate change, such as increased temperature or extreme weather events, are the cause of diseases, for example air pollution (from forest fires), or water pollution (from floods). Likewise, these effects can lead to direct injuries from extreme events or chronic malnutrition by reducing agricultural production. Which can even increase vector-borne infections, such as dengue fever. In addition, the loss of infrastructure in health services, political instability and poor climate governance amplify these risks and significantly threaten the health and well-being of the Peruvian population.

In 2015, 193 countries joined the Paris Climate Agreement, committing to develop actions to limit global warming to less than 2°C in global annual average temperature, and preferably to less than 1.5°C, compared to pre-industrial temperatures. This 1.5°C target is crucial, as it represents a key tipping point ^1^. If we exceed this limit, the chances of suffering the effects of climate change, such as extreme floods, droughts, forest fires and food shortages, could increase dramatically.

It is important to recognize that reversing the increasing global temperature is extremely challenging, even if the greenhouse gas emissions are reduced, given that our planets’ global average is already 1.15°C warmer than during the preindustrial age. The World Meteorological Organization has predicted that, in the next five years, the combination of the El Niño phenomenon and climate change will rise global temperatures into unfamiliar territory, which means rising above the critical value of 1.5°C ^2^.

Peru is responsible of 0.38% of global greenhouse gas emissions ^3^. Even if this value may seem relatively small, it is not proportional to the impact of climate change in our country. National policies, such as the Nationally Determined Contributions (NDC) and the National Adaptation Plans (NAP), should place the health of the populations as the main focus of decision-making. Therefore, the Peruvian Plan of National Adaptation to Climate Change should include preventive adaptation measures that answer to gradual changes related to heat, infectious diseases, forest fires, droughts and floods, as well as preparation for events as extreme as El Niño. We have to create climate-resilient health systems, implement climate observatories with real-time health and weather information, a national early warning system for extreme heat, and increase the generation of green spaces such as parks and “green roofs” in cities, in order to improve the national response to extreme heat events ^4^.

The Peruvian population is facing the impact of several events related to climate change, such as extreme heat, which is the most important. According to The Lancet Global Countdown 2022 report, between 2017 and 2021, Peruvians were exposed to mean summer temperatures that were on average 0.2°C higher than those recorded in the time frame between 1986 and 2005 ^5^. However, this increase varies by region due to the different microclimates. A consistent and prolonged temperature increase (0.8°C higher than the annual average) has been reported in at least eight Peruvian regions (Áncash, Cajamarca, Huánuco, Junín, La Libertad, Lambayeque, Piura and Tumbes), compared to the period between 1960 and 1980. This temperature increase is also related to an increase in premature deaths, particularly in vulnerable populations. In this sense, a 152% increase in heat-related mortality has been observed in adults over 65 years of age, throughout Peru, when comparing the periods of 2000-2004 and 2017-2021 ^6^. Additionally, increasing temperatures have significant implications in the incidence of vector-borne infectious diseases, such as dengue, due to the increase in climatic suitability (ideal climatic conditions) for the transmission of vector-borne diseases. In fact, data from the Lancet Countdown Global 2022 report reveals that between 2012 and 2021, the R0 of *A. aegypti* and *A. albopictus* increased by 0.83 and 1.14, respectively, compared to the baseline period (1951-1960) ^5^. These higher values indicate that the disease is spreading more easily and quickly, which may result in more dengue outbreaks in areas that were previously free of the disease.

Mitigation strategies, such as decarbonization, which prioritizes public transport and using bicycle, benefit health and the environment by reducing the consumption of fossil fuels and pollution. To achieve these goals, it is essential to promote access to safe, affordable and reliable public transport networks, which would also reduce socioeconomic inequalities associated with transport. Currently, Lima is the most polluted city in South America, with an average fine particle (PM_2.5_) concentration of 26 µg/m^3 (^^7^, which exceeds the ​ annual average recommended by the World Health Organization (WHO) of 5 µg/m^3^. PM_2.5_ is one of the most dangerous environmental pollutants for human health due to its size, which allows it to penetrate deep into the lungs. High concentrations of this pollutant can aggravate chronic, heart, lung and mental diseases, as well as increase hospital visits due to acute episodes and, in many cases, premature death. In Peru, during 2019, 7,800 deaths were attributed to air pollution by PM_2.5_ and approximately one third of these deaths were directly related to the burning of fossil fuels ^5^. According to The Lancet Global Countdown 2022 report, the monetary cost of these premature deaths due to pollution is equivalent to 1.4% of Peru’s gross domestic product (GDP) in 2021, which represents the average income of almost half a million Peruvians ^5^.

These data underline the urgency of addressing the impact of climate change on public health in Peru with comprehensive strategies that encompass adaptation to extreme temperatures and improvement of air quality. By prioritizing these aspects, the well-being and health of Peruvians can be protected, social inequalities reduced, and a sustainable and resilient future guaranteed for all. It is essential to develop comprehensive strategies that address the multidimensional challenges posed by climate change and that prioritize the protection of public health. The implementation of effective measures is required to reduce greenhouse gas emissions, promote sustainable practices in key sectors and strengthen the response and adaptation capacity of communities in the face of the impact of climate change. In addition, it is essential to improve climate governance, since it plays a vital role in the effective response to the challenges of climate change. Multi-level climate governance that involves coordination and collaboration among various actors, including the public sector, local governments, the private sector, civil society, and academia is needed. The governance framework should promote collaboration, articulate policies and plans at the local level, and facilitate the implementation of adaptation measures. In addition, decentralization should be encouraged in order to allow local and regional governments to develop adaptation plans specific to their contexts.

These proposed changes in our actions and in our decision-making will not only prepare us to better respond to the effects of climate change, but will also advance the well-being of all Peruvians. We already have the evidence we were looking for, now it’s time to act.
