# Supplementary material for: Impact of climate change on the health of peruvians: challenges and strategies for a comprehensive response
Source: Rev Peru Med Exp Salud Publica. 2023 Jun 30;40(2):130–1. doi: 10.17843/rpmesp.2023.402.12998 (PMC10953657; doi:10.17843/rpmesp.2023.402.12998)
Supplement: Translation

# Translation: Impacto del cambio climático en la salud de los peruanos: desafíos y estrategias para una respuesta integral

**Authors:** Luciana Blanco-Villafuerte, Stella M. Hartinger

**Affiliations:** 1 Centro Latino Americano de Excelencia en Cambio Climático y Salud, Universidad Peruana Cayetano Heredia, Lima, Perú.

El cambio climático está ocasionando impactos negativos en la salud de todas las poblaciones, incluyendo la población peruana. Los efectos del cambio climático, como el aumento de la temperatura o los eventos meteorológicos extremos están causando enfermedades, por ejemplo a través de la contaminación atmosférica de partículas (por incendios forestales), o la contaminación de agua (por inundaciones). Así mismo, los efectos pueden generar lesiones directas por estos eventos extremos o desnutrición crónica al reducir la producción agrícola. Incluso puede aumentar las infecciones transmitidas por vectores, como es el caso del dengue. Además, la pérdida de infraestructura en los servicios de salud, la inestabilidad política y la pobre gobernanza climática amplifican estos riesgos y amenazan la salud y el bienestar de la población peruana de manera significativa.

En 2015, 193 países se unieron en el Acuerdo Climático de París, comprometiéndose a desarrollar acciones para limitar el calentamiento global a menos de 2 °C, en el promedio anual global de temperatura, y preferiblemente a menos de 1,5 °C, en comparación con las temperaturas preindustriales. Este objetivo de 1,5 °C es crucial, ya que representa un punto de inflexión clave ^1^. Si superamos este límite, las posibilidades de sufrir los efectos del cambio climático, como inundaciones extremas, sequías, incendios forestales y escasez de alimentos podrían aumentar drásticamente.

Es importante reconocer que revertir por completo el incremento de las temperaturas globales es extremadamente desafiante, incluso si reducimos las emisiones de gases de efecto invernadero, dado que el planeta ya se encuentra aproximadamente 1,15 °C más caliente en promedio global que en la era preindustrial. Dicho esto, la Organización Meteorológica Mundial ha predicho que, en los siguientes cinco años, la combinación del Fenómeno de El Niño y el cambio climático va a llevar a las temperaturas globales a un territorio desconocido, lo que significa superar temporalmente el umbral crítico de 1,5°C ^2^.

El Perú es responsable de 0,38% de las emisiones globales de gases de efecto invernadero ^3^. Aunque esta cifra puede parecer relativamente pequeña, no es proporcional al impacto que ya está generando el cambio climático en nuestro país. Es fundamental que las políticas nacionales, como las Contribuciones Nacionalmente Determinadas (NDC, por sus siglas en inglés) y los Planes Nacionales de Adaptación (NAP, por sus siglas en inglés), coloquen la salud de nuestras poblaciones en el centro de la toma de decisiones. Por esto es importante que el Plan Nacional de Adaptación al Cambio Climático de Perú implemente medidas de adaptación preventivas que respondan a cambios graduales vinculados al calor, enfermedades infecciosas, incendios forestales, sequías e inundaciones así como prepararse para eventos extremos como el Fenómeno de El Niño. Tenemos que crear sistemas de salud resilientes al clima, implementar observatorios climáticos con información a tiempo real de salud e información meteorológica, un sistema nacional de alerta temprana para calor extremo y aumentar la generación de espacios verdes como parques y «techos verdes» en las ciudades, para mejorar la respuesta a eventos extremos de calor ^4^.

Entre los diferentes impactos que la población peruana ya experimenta por el cambio climático, el calor extremo es uno de los más importantes. Según el informe The Lancet Countdown Global 2022, entre el 2017 y 2021, los peruanos estuvieron expuestos a temperaturas medias de verano que eran en promedio 0,2 °C más altas que las registradas en la ventana de tiempo entre 1986 y 2005 ^5^. Sin embargo, este aumento varía según región debido a la presencia de microclimas en el país. Al menos, en ocho regiones del Perú (como Áncash, Cajamarca, Huánuco, Junín, La Libertad, Lambayeque, Piura y Tumbes), se ha observado un incremento consistente y prolongado de las temperaturas, llegando a aumentar en promedio 0,8 °C (promedio anual) en comparación con el período de 1960 y 1980. Este aumento de temperaturas también está relacionado con un incremento en las muertes prematuras, especialmente en personas vulnerables. En ese sentido, se ha observado un incremento del 152% en la mortalidad relacionada con el calor en adultos mayores de 65 años, de todo el Perú, comparando los períodos de 2000-2004 y 2017-2021 ^6^. Adicionalmente, el incremento térmico tiene implicaciones significativas en la incidencia de enfermedades infecciosas transmitidas por vectores, como el dengue, debido al incremento de la idoneidad climática (condiciones climáticas ideales) para la transmisión de enfermedades transmitidas por vectores. De hecho, los datos del informe Lancet Countdown Global 2022 revelan que entre 2012 y 2021, el R0 de *A. aegypti* y *A. albopictus* aumentó en 0,83 y 1,14, respectivamente, en comparación con el período base (1951-1960) ^5^. Estos valores más altos indican que la enfermedad se está propagando más fácil y rápidamente, lo que puede dar como resultado un aumento de los brotes de dengue en zonas que antes no tenían esta enfermedad.

Las estrategias de mitigación, como la descarbonización, en la cual se priorice el transporte público y el uso de bicicletas, benefician la salud y el medioambiente al reducir el consumo de combustibles fósiles y la contaminación. Para lograrlo, es fundamental promover el acceso a redes de transporte público seguras, asequibles y confiables, lo que también disminuiría las desigualdades socioeconómicas asociadas al transporte. Actualmente, Lima se posiciona como la ciudad más contaminada de Sudamérica, con un promedio de concentración de partículas finas (PM_2.5_) de 26 µg/m^3 (^^7^, lo cual supera los valores recomendados por la Organización Mundial de la Salud (OMS) de 5 µg/m^3^ de promedio anual. El PM_2.5_ es uno de los contaminantes ambientales más peligroso para la salud humana debido a su tamaño, que le permite penetrar profundamente en los pulmones. Su presencia en altas concentraciones agrava las enfermedades crónicas, cardíacas, pulmonares y mentales, aumenta las visitas hospitalarias por sus episodios agudos y, en muchos casos, puede ocasionar la muerte prematura. En el Perú, durante el 2019, se atribuyeron 7800 muertes a la contaminación del aire ambiental por PM_2.5_ y aproximadamente un tercio de estas muertes estuvieron directamente relacionadas con la quema de combustibles fósiles ^5^. Según el informe The Lancet Countdown Global 2022, el costo monetario de estas muertes prematuras debido a la contaminación equivale al 1,4% del producto interno bruto (PIB) del Perú en el 2021, lo que representa los ingresos medios de casi medio millón de peruanos ^5^.

Estos datos subrayan la urgencia de abordar el impacto del cambio climático en la salud pública de Perú con estrategias integrales que abarquen la adaptación a las temperaturas extremas y la mejora de la calidad del aire. Al priorizar estos aspectos, se puede proteger el bienestar y la salud de los peruanos, reducir las desigualdades sociales y garantizar un futuro sostenible y resiliente para todos. Es esencial desarrollar estrategias integrales que aborden los desafíos multidimensionales planteados por el cambio climático y que prioricen la protección de la salud pública. Se requiere la implementación de medidas efectivas para reducir las emisiones de gases de efecto invernadero, promover prácticas sostenibles en sectores clave y fortalecer la capacidad de respuesta y adaptación de las comunidades frente al impacto del cambio climático. Además, es fundamental mejorar la gobernanza climática. Ya que esta, desempeña un papel vital en la respuesta efectiva a los desafíos del cambio climático. Se necesita una gobernanza climática multinivel que involucre la coordinación y colaboración entre diversos actores, incluidos el sector público, los gobiernos locales, el sector privado, la sociedad civil y la academia. Este marco de gobernanza debe promover la colaboración, articular políticas y planes a nivel local, y facilitar la implementación de acciones de adaptación. Además, se debe fomentar la descentralización para permitir que los gobiernos locales y regionales desarrollen planes de adaptación adaptados a sus contextos específicos.

Estos cambios propuestos en nuestro accionar y en nuestra toma de decisiones no solo logrará prepararnos y responder mejor a los efectos del cambio climático, sino también avanzar en el logro del bienestar de todos los peruanos. Ya tenemos la evidencia que buscábamos, es momento de actuar.
